# The impact of COVID-19 national restrictions on dental antibiotic dispensing trends and treatment activity in England: January 2016 to July 2021

**DOI:** 10.1093/jacamr/dlad081

**Published:** 2023-07-17

**Authors:** Angela Falola, Alicia Demirjian, Wendy Thompson, Colin S Brown, Sarah Gerver, Sabine Bou-Antoun

**Affiliations:** HCAI, Fungal, AMR, AMU & Sepsis Division, United Kingdom Health Security Agency (UKHSA), London NW9 5EQ, UK; HCAI, Fungal, AMR, AMU & Sepsis Division, United Kingdom Health Security Agency (UKHSA), London NW9 5EQ, UK; Department of Paediatric Infectious Diseases & Immunology, Evelina London Children’s Hospital, London SE1 7EH, UK; Faculty of Life Sciences & Medicine, King’s College London, London WC2R 2LS, UK; Division of Dentistry, University of Manchester, Manchester M13 9PL, UK; HCAI, Fungal, AMR, AMU & Sepsis Division, United Kingdom Health Security Agency (UKHSA), London NW9 5EQ, UK; NIHR Health Protection Research Unit in Healthcare Associated Infections and Antimicrobial Resistance, Imperial College London, London, UK; HCAI, Fungal, AMR, AMU & Sepsis Division, United Kingdom Health Security Agency (UKHSA), London NW9 5EQ, UK; NIHR Health Protection Research Unit in Healthcare Associated Infections and Antimicrobial Resistance, Imperial College London, London, UK; HCAI, Fungal, AMR, AMU & Sepsis Division, United Kingdom Health Security Agency (UKHSA), London NW9 5EQ, UK

## Abstract

**Introduction:**

Reducing inappropriate antibiotic prescribing tempers the growing threat of antimicrobial resistance. We aimed to quantify the associated impact of COVID-19-related national restrictions in England on dental antibiotic dispensing and describe changes in appointments and modes of delivery of care.

**Methods:**

Interrupted time series analyses were completed using NHS Business Service Authority (NHSBSA) ePACT2 data to measure the associated change in antibiotic dispensing in England following COVID-19-related restrictions (which began March 2020). For face-to-face dental consultations, NHS dental treatment plan (FP17) data were used. For remote consultations during the COVID-19 period, NHSBSA Compass system remote management data were used.

**Results:**

Between January 2016 and February 2020, there was a decreasing trend in antibiotic dispensing (−0.02 per 1000 population per month, *P *< 0.05). In contrast, there was an increase of 0.98 per 1000 population (*P* < 0.05) in March. The peak in antibiotic use occurred between June 2020 and July 2020, once the restrictions were eased. At the end of the study period (July 2021), the elevated prescribing trend had not returned to pre-pandemic counterfactual levels, although exhibiting a declining trend. A stable trend in dental treatment plans was seen pre-COVID-19, with a sharp decline coinciding with the restrictions. Dental treatment plans had not yet returned to the higher pre-pandemic levels.

**Conclusions:**

Dental antibiotic prescribing significantly increased with the national COVID-19 restrictions when service delivery was altered with the closure of dental practices and introduction of remote consultations. Teledentistry was likely associated with inappropriate antibiotic prescribing. Continued antimicrobial stewardship and prudent use of antibiotics in dentistry is important.

## Introduction

Efforts to tackle the growing threat of antimicrobial resistance through prudent antimicrobial prescribing has seen usage of antibiotics within primary care steadily decrease in England over the past decade. In England, primary care accounts for approximately 81% of antibiotic consumption and dental practice accounts for 9% of consumption within primary care.^1^ Despite the intense strain that the SARS-CoV-2 coronavirus (causing COVID-19 disease) pandemic had on healthcare systems, the level of antibiotic prescribing in primary care continued to decrease in England, and across the EU, with evident unprecedented declines.^1–4^

This decrease in antibiotic usage within primary care was not seen within the dental setting, where there was a marked concurrent rise in antibiotic prescribing during the months following the first ‘lockdown’ (a national measure encouraging social isolation) in England (March 2020–May 2020).^5,6^ The COVID-19 pandemic and first ‘lockdown’ period in England altered dental practice services and prescribing, with the closure of dental practices for routine, elective and non-urgent care, and only urgent or emergency dental care made available during this period.^6,7^ Service delivery changed with new and accelerated use of remote consultations, either over the phone or online, and a departure from the usual face-to-face consultation.^8^ Remote consultations and triage services were used with the aim of delivering dental service via advice, analgesia and antimicrobials (AAA), where appropriate; alternatively, a referral was made when absolutely necessary and when treatment could not be delayed.^5^ Treatment was mainly available through NHS urgent dental regional hubs and centres.^6,9^

With the national restriction in access to dental care and the implicated change in service delivery during the COVID-19 ‘lockdown’ we primarily aimed to quantify the correlated impact of this on antibiotic dispensing in the dental setting in England. We also aimed to descriptively assess the impact of restricted dental access on changes in the mode of treatment activities (face-to-face treatment plans and remote consultations) and whether this altered antibiotic prescribing behaviour.

## Methods

### Data sources

Between 1 January 2016 and 31 July 2021, antibiotic dispensed data were used from NHS dental drug prescription FP10 (D) forms obtained from ePACT2, NHS Business service Authority (NHSBSA). Data included antibiotic items dispensed by community pharmacists to NHS dental services patients in England and excluded prescriptions from prisons, hospitals and the private sector. Antibiotics included within the study were from the British National Formulary (BNF) Chapter 5.1: antibacterial drugs; and doxycycline hyclate from BNF Chapter 12.3.1.

Data related to dental treatment plans were obtained from NHS dental treatment plan (FP17) forms.^10^ Other than during the COVID-19 pandemic, data relating to remote dental consultations/management have never been routinely collected by NHS England. Remote consultation data were collected during the COVID-19 pandemic on the NHSBSA Compass system^11^ and as part of its system of paying dentists during the period from 13 May 2020 to 31 July 2021 (validated from 13 May 2020). The NHSBSA Compass system also provided details on primary reasons for antibiotic dispensing for these remote consultations.^10,11^ England population data were obtained from the Office for National Statistics.^12^ The mid-year population estimates for 2020 were used as a proxy for 2021 rates.

### Data analysis

Monthly total dental antibiotic prescribing rates were calculated as number of items per 1000 population dispensed by community pharmacists to NHS dental services patients in England. Similarly, the rate of face-to-face treatment plans were estimated by the number of face-to-face treatment plans per 1000 population and remote consultation per 1000 population.

A quasi-experimental, segmented regression of interrupted time series was used to assess the correlated impact of COVID-19 restrictions on antibiotic consumption (total antibiotic items per 1000 population). Monthly data for the study period were assessed, including 50 months pre-implementation of national restrictions (i.e. the intervention; implemented March 2020) and 17 months post-implementation period. To account for autocorrelation within the data, a seasonally adjusted autoregressive moving average (ARMA) model was fitted. The underlying trend before the COVID-19 national restrictions was used to predict the expected trend post-national restrictions, in the absence of COVID-19 (the counterfactual). This counterfactual acts as the control or comparator for the observed trends following the COVID-19 national restrictions. The order of the moving average and the autoregressive model parameters were determined using scatter plots of the deviance residuals versus time, the Durbin–Watson test and the autocorrelation and partial autocorrelation functions.^13–15^ To assess the fit of the model parameters, the maximum-likelihood ratio test and quantile–quantile plots were used.^15^ Statistical significance was attributed for *P *< 0.05.

## Results

### Antibiotic prescribing changes

**Figure 1.**
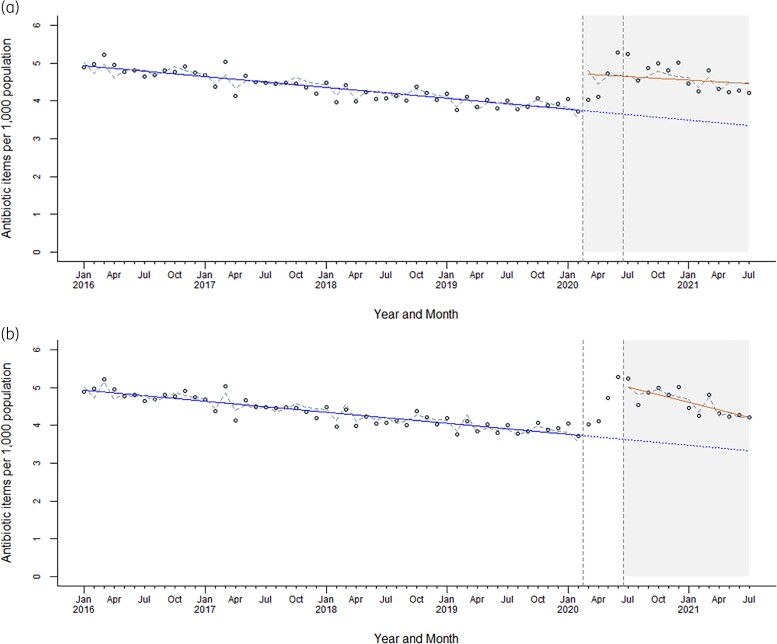
Dispensed dental antibiotic items per 1000 population in England, January 2016 to July 2021, with observed pre-COVID-19 trend and interrupted time series analyses. (a) Interrupted at the time of first England restrictions and first COVID-19 wave (March 2020). (b) Lagged impact with interruption at phased lifting of restrictions (July 2020).

Between 2016 and 2019 there have been consistent annual reductions in antibiotic dispensing (−18.8% over the 4 years). The COVID-19 pandemic and the resulting restrictions on dental services across England caused major disruptions to dental services and, as such, on dental prescribing. Despite the decrease in dental treatment activities since March 2020, there was a marked rise in total antibiotic items dispensed during the pandemic period in England (pre-March 2020 mean items per 1000 population was 4.3 and post-March 2020 was 4.6, with increases in the rate of dispensing visibly beginning in May 2020 and peaking in June 2020 (increase of 39.4% items per 1000 population between June 2019 and June 2020, from 3.8 to 5.3 antibiotic items per 1000 population) (Figure 1a).

Interrupted time series analysis (Figure 1a, Table 1) confirmed a slight decreasing trend in antibiotic dispensing pre-COVID-19 dental service restrictions (by 0.02 per 1000 population per month, *P *< 0.05). This reversed with the introduction of national restrictions in March 2020, with a coinciding 26.7% relative incline in the rate of antibiotic dental dispensing (equating to 0.98 items per 1000 population level increase, *P *< 0.05). A sensitivity analysis using a lag period in the interruption, applied to July 2020, was completed to assess the impact of national easing of restrictions (Figure 1b). This period (July 2020) coincided with the greatest incline in antibiotic dispensing observed, equating to 1.44 items per 1000 population (*P *< 0.05), followed by a declining trend (Figure 1b, Table 1). As of the end of the study period (July 2021) dental antibiotic dispensing had not returned to pre-pandemic counterfactual levels; the absolute change suggested that the average monthly antibiotic dispensing rate was 1.07 per 1000 population greater than would have been expected had the COVID-19 social restrictions not been implemented, representing a 31.3% increase relative to what would have been expected had the existing trend continued (Table 1).

**Table 1.** Findings from the interrupted time series analyses on antibiotic prescribing (items per 1000 population)

Intervention period measured	Estimate of intercept (*P* value)	Pre COVID-19 restrictions prescribing trend (*P* value)	Change in prescribing level (*P* value)	Change in post-COVID-19 restriction prescribing trend (*P* value)	Absolute change in prescribing, July 2021	Relative change (%), July 2021
(a) Feb–Mar 2020: social restrictions	5.03 (<0.0001)	−0.024 (<0.0001)	0.98 (<0.0001)	0.008 (0.5697)	1.07	31.34
(b) Jun–Jul 2020: easing restrictions	5.03 (<0.0001)	−0.025 (<0.0001)	1.44 (<0.0001)	−0.042 (0.0002)	0.74	21.16

### Face-to-face treatment plans (FP17 forms)

**Figure 2.**
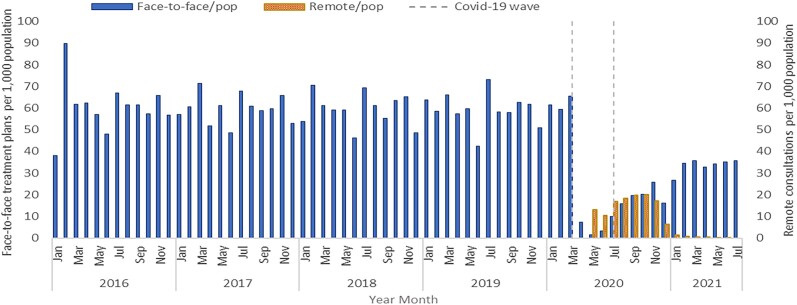
Dental patient management measured by face-to-face treatment plans per 1000 population (pop) and remote consultations per 1000 population in England, January 2016 to July 2021.

Prior to the introduction of restrictions (26 March 2020, coinciding with the first COVID-19 wave) in England, there was a slightly decreasing trend in the rate of dental face-to-face treatment plans (FP17 forms per 1000 population), with a total reduction of 2% over the 4 year period pre-pandemic (Figure 2). Between 2019 and 2020 there was a sharp decline in dental face-to-face treatment plans (FP17 forms) of 56.9%. The greatest decrease began in April 2020, with a reduction of 88.8% between March 2020 and April 2020 (from 65.5 to 7.4 face-to-face treatment plans per 1000 population) and a reduction of 87.2% between April 2019 and April 2020 (from 57.3 to 7.4 in dental face-to-face treatment plan per 1000 population). This initial face-to-face management decrease observed in April 2020 was followed by the introduction and increase in dental remote consultations from May until July 2020, when dental service restrictions began to ease in England. The easing of national restrictions saw an increase in face-to-face management, alongside continued remote management (Figure 2). By January 2020 and until the end of the study period (July 2021), remote consultations had substantially reduced; however dental face-to-face management had not returned to pre-pandemic levels by 31 July 2022 (Figure 2).

### Remote contacts (Compass system) per 1000 population

Prior to the COVID-19 pandemic and dental service restrictions being imposed there were no remote dental contacts recorded. During the first COVID-19 wave in England and at the onset of the national ‘lockdown’ period, service delivery altered with not only a vast decrease in face-to-face treatment plans in April 2020, but a shift with the introduction of remote contact in May 2020 (remote contact accounting for 90% of dental contacts in May 2020) (Figure 2). The highest rate of remote contacts was in October 2020, with 20.1 remote consultations per 1000 population occurring.

### Remote consultations: primary reason for items dispensed (Compass system)

**Figure 3.**
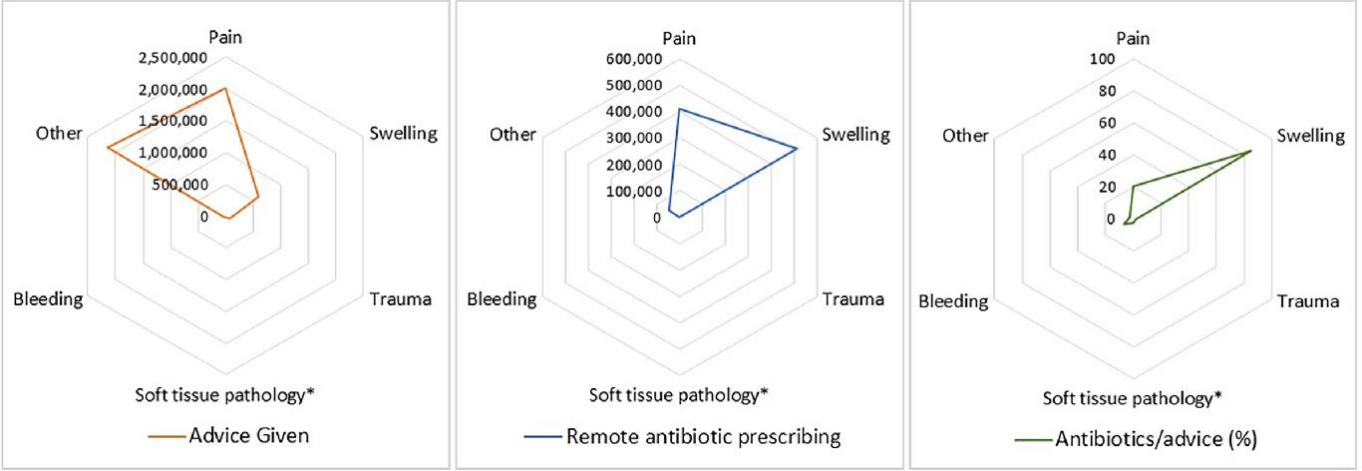
Primary reason behind remote consultation dental advice given and for remote antibiotic prescriptions in England, May 2020 to June 2021. (*An example of soft tissue pathology would be suspected oral cancer.)

The NHSBSA Compass system provided details on the primary reason recorded for remote triaging and management between May 2020 and June 2021. The most common primary reason patients sought advice during this period were for indications of pain (Figure 3). Antibiotics were most commonly prescribed for primary reasons of swelling, with 53% of total antibiotics prescribed within remote management reporting this as a primary reason, followed by pain (42%). Bleeding, trauma and soft tissue pathology as primary reasons resulted in very low antibiotic prescribing (<0% combined of total). Although a large proportion of total antibiotic prescriptions were for pain, when percentage of antibiotics prescribed were calculated by the number of times advice was given for the primary indications, 20% of those for whom advice for pain was the primary reason were prescribed antibiotics compared with 85% for swelling.

## Discussion

The analysis within this study provides the first quantification of the impact of COVID-19 national restrictions on antibiotic prescribing, and the first known description of the changes in mode of delivery of care within dental practices in England during this period.

Between January 2016 and February 2020, antibiotic dispensing had been slowly decreasing; however, this trend was reversed with implementation of national COVID-19 restrictions and closure of dental practices, with a level increase of 0.98 in items per 1000 population in March 2020 (*P *< 0.05). The peak of the incline (1.44 items per 1000 population level increase) was evident between June 2020 and July 2020, coinciding with a phased lifting of national restrictions and when dental practices began to reopen (8 June 2020). There were subsequent declining trends from this point to the study end date of 31 July 2021. Interestingly, the data suggest continued recording of remote services for 6 months after this point. Notably, although the level of dispensing since dental practices reopened (June 2020) is once again on a downward trend, it has not returned to the pre-pandemic counterfactual levels.

The findings from this study are corroborated with increases seen in dental antibiotic dispensing coinciding with COVID-19 dental service provision restrictions in Scotland (by 49%), France (17.9%), Belgium (18.3%), New York and Georgia (USA; 6.5% and 7.7%, respectively) and Alberta (Canada; 76%).^16–20^ In contrast, Australia (−16%) and Ontario (Canada; −12.3%) experienced a decrease in dental antibiotic prescribing following their COVID-19 restrictions. The Australian study, however, reported a 20% increase in broad-spectrum antibiotics shortly after (a comparison of June 2019 and June 2020) which, similarly to the increases noted once restrictions were eased within this study, may be related to addressing a backlog of demand in dental services, or perhaps reflective of delayed treatment and clinical deterioration of patients.^21^ A growth in patients accessing face-to-face dental care was not demonstrated within our findings; although there was an increasing trend in face-to-face treatment plans with the easing of national restrictions, the trend had not returned to pre-pandemic treatment plans or appointment levels within NHS dental practices.

The initial increase in dental antibiotic dispensing following the national dental service restrictions is multifactorial. The literature suggests that increasing antibiotic prescribing is influenced by dental practitioners finding it challenging to correctly diagnose patients remotely with lack of physical clinical assessment; dentists may also be put under pressure by patients to prescribe antibiotics, or perceived patient expectations.^22–24^ Dentists may also have had concerns of progression of a bacterial infection and deterioration where these infections may otherwise be missed without thorough face-to-face assessment.^23,25^ It has also been suggested that urgent dental centres, which remained available for emergency dental care, although themselves having limited capacity related to workforce redeployment to other essential services,^26^ had a prerequisite that patients should have firstly been prescribed antibiotics if needed prior to referral for face-to-face treatment.^5,23,24^

UK dental guidelines indicate that the majority of dental infections are amenable to dental intervention and operative procedures, meaning the use of antibiotics is often inappropriate.^24^ The COVID-19 restrictions, and subsequent cessation of face-to-face management for patients with acute dental problems, meant that dentists were unable to treat patients with acute dental pain or infection as set out by the guidelines. Our findings suggest that swelling and pain were the most common primary reasons for antibiotics being dispensed. Although prescribing for pain as a primary reason may be indicative of inappropriate antibiotic prescribing, findings from a recent survey suggested that dentists were concerned with the inaccurate description of acute pain by patients and were less confident diagnosing and treating patients with acute pain remotely,^24^ demonstrating the importance of physical examination within the dental setting to avoid inappropriate prescribing^24^. Other healthcare settings displayed decreases in antibiotic prescribing in England. General practices, which were also restricted to mostly remote consultation and faced similar challenges during the first national ‘lockdown’ restrictions, displayed trends with substantial decreased antibiotic dispensing.^1,27^ This may be related to general practices not always requiring intervention and exemplifies the limitations of remote management within the dental setting. The increased prescribing once restrictions eased suggests the lack of local urgent dental care centre support/capacity available and the limitations of AAA during a pandemic.

During the first COVID-19 wave and dental care restriction period in England, all patients in England who required dental care were treated within the NHS. With the easing of restrictions, private dental providers would have increasingly offered their services. Private dental data, which account for approximately 25% of primary care dental care in England,^24^ were not included within the datasets used. Hence, treatment plans and antibiotic prescribing from June 2020 onwards are underestimated, and this is a limitation of the data used.^5^ A further limitation to be noted is that although we were able to assess the primary reason for remote antibiotic prescriptions, it was not possible to determine appropriateness of prescriptions or indications for antibiotic prescribing. Furthermore, we were unable to differentiate between classes of antibiotics dispensed remotely with a lack of data on this. To provide a more nuanced understanding of the impact of COVID-19 national restriction on treatment activities, it would be advantageous to assess antibiotic classes dispensed, both face to face and remotely.

In the wake of the COVID-19 pandemic, there is a need to review antibiotic stewardship within the dental setting, to improve knowledge and research of remote dental patient management to inform guidance for this mode of delivery of care, as well as preserving access of face-to-face dental care in future pandemics for certain indications such as pain (e.g. toothache) to avoid inappropriate antibiotic prescribing, subsequent implications on antimicrobial resistance and future increased patient expectations for antibiotics for clinically inappropriate indications.^1^ As the level of face-to-face treatment plans has declined during the pandemic it is important for dental practices to provide a system for patients to access services and capacity within the practices and centres to be able to do so. There are also concerns around equity of access of remote management.^16,28,29^ It would be beneficial to examine remote antibiotic items prescribed by age, ethnicity and measures of social deprivation as there are concerns that the shift to telemedicine may have exacerbated disparities in health access.^30^

### Conclusions

In conclusion, our findings showed that dispensing of NHS dentalprescriptions increased following the introduction of national dental care restrictions associated with the COVID-19 pandemic in England. This is likely related to changes in the mode of dental treatment provision to remote consultations. The primary reason for remote antibiotic dispensing was frequently documented for pain, indicating inappropriate prescribing with teledentistry.
